# Estrogen-mediated DNMT1 and DNMT3A recruitment by EZH2 silences miR-570-3p that contributes to papillary thyroid malignancy through DPP4

**DOI:** 10.1186/s13148-024-01685-z

**Published:** 2024-06-18

**Authors:** Xiarong Hu, Qingyao Ye, HuanQuan Lu, Zhiming Wu, Siyuan Chen, Ruinian Zheng

**Affiliations:** 1 Department of General Surgery, The Tenth Affiliated Hospital of Southern Medical University (Dongguan People’s Hospital), Dongguan, 523059 Guangdong China; 2 Department of Oncology, Dongguan Institute of Clinical Cancer Research, Dongguan Key Laboratory of Precision Diagnosis and Treatment for Tumors, The Tenth Affiliated Hospital of Southern Medical University (Dongguan People’s Hospital), Dongguan, 523059 Guangdong China

**Keywords:** Papillary thyroid carcinoma, Estrogen, miR-570-3p, DPP4, Methylation

## Abstract

**Background:**

Papillary thyroid carcinoma (PTC) is a common endocrine malignancy. Studies have indicated that estrogen can regulate the expression of miRNAs in numerous malignancies. MiR-570-3p has been shown to have a regulatory function in various cancers. However, studies of the regulatory function of miR-570-3p and a direct link between estrogen (especially estradiol E2) and miR-570-3p in PTC have not been done.

**Methods:**

Expression of miR-570-3p and its downstream target DPP4 in PTC tissues and cells was predicted using bioinformatics and validated by qRT-PCR and western blot assays. We then performed a series of gain-and-loss experiments to assess the functional significance of miR-570-3p/DPP4 axis in PTC progression in vitro and in vivo. Additionally, the methylation of the miR-570-3p promoter region was examined via bioinformatics analysis and MSP. Finally, the effects of E2 on PTC progression and the correlation between DNMT1/DNMT3A and EZH2 were predicted by bioinformatic tools and proved by luciferase reporter, ChIP, and co-IP assays.

**Results:**

In PTC tumor tissues and cell lines, there was a lower expression level and a higher methylation level of miR-570-3p compared to normal tissues and cell lines. DPP4 was identified as the downstream target of miR-570-3p. Overexpression of miR-570-3p reduced the proliferative, migratory, and invasive capabilities, and promoted apoptosis, while overexpression of DPP4 reversed these effects in PTC cells. It was also discovered that DNMT1 and DNMT3A increased the CpG methylation level of the miR-570-3p promoter in an EZH2-dependent manner, which led to decreased expression of miR-570-3p. Furthermore, we observed that estrogen (E2) enhanced the methylation of miR-570-3p and suppressed its expression levels, resulting in augmented tumor growth in vivo in PTC*.*

**Conclusion:**

Estrogen regulates the EZH2/DNMTs/miR-570-3p/DPP4 signaling pathway to promote PTC progression.

## Introduction

Thyroid cancer (TC) is one of the most common endocrine malignancies worldwide [1]. Recently, the incidence of TC has increased rapidly, particularly among young women [2]. Papillary thyroid carcinoma (PTC) accounts for approximately 85% of all TC cases [3]. Generally, the prognosis for PTC is favorable due to its slow growth rate and the retention of iodide uptake [4]. However, a significant number of patients experience invasiveness and distant metastasis of PTC. Research has indicated that PTC tumorigenesis is influenced by various factors, including gene mutations and epigenetic changes [5]. Therefore, there is an urgent need to understand better the molecular mechanisms involved in the pathogenesis of PTC.

The high incidence of PTC in women suggests that estrogen-related signaling pathways may exert a crucial function in PTC development [2, 6]. Estrogen mainly regulates its target gene expression via activating ER-α and ER-β [7]. Estradiol (E2) is a common estrogen and has been confirmed to be involved in physiological and pathological processes, including PTC progression [8]. E2 exerts the regulatory function via activating the ER-α-mediated signaling pathway, which is involved in the modulation of cell proliferation and migration [9]. Importantly, estrogen has been proven to modulate different miRNAs in cancer [10].

MicroRNAs (miRNAs) are non-coding RNAs of small molecular size that can facilitate mRNA degradation or suppress mRNA translation [11]. MiRNAs take part in multiple cellular processes, including proliferation, apoptosis, and migration [12]. Several studies have confirmed the dysregulation of miRNAs in different types of human cancers [13–15]. MiR-570-3p has been identified as being abnormally expressed in various organs and plays a regulatory role in human cancers [16, 17]. Yue et al. suggested that miR-570-3p can inhibit breast cancer progression and reduce doxorubicin resistance [18]. Bao et al. revealed that metformin promotes miR-570-3p expression via demethylation, thereby repressing metastasis and autophagy in osteosarcoma [19]. In colon cancer, miR-570-3p has been shown to function as a tumor suppressor [20]. In bladder cancer, miR-570-3p is reported to be the target of circFUT8 [21]. However, the role of miR-570-3p in the context of PTC pathogenesis has not yet been investigated.

DNA methyltransferases (DNMTs) contribute to increased methylation at specific loci by adding methyl groups to the cytosine residues in the DNA, typically at CpG dinucleotides. This process can lead to transcriptional silencing of genes, particularly when it occurs in gene promoter regions [22]. Examples of such DNMTs include DNMT1, DNMT2, DNMT3A, DNMT3B and DNMT3L [23]. Among these, DNMT1, DNMT3A, and DNMT3B are the most typical C5 DNMTs. DNMT1 is responsible for maintaining methylation, while DNMT3A and DNMT3B are involved in de novo methylation [24]. EZH2 is a catalytic subunit of the PRC2 complex and can serve as a recruitment platform for DNMTs to promote transcriptional repression of target genes [25, 26]. EZH2 interacts with DNMTs within the context of the Polycomb repressive complexes 2 and 3 [27]. It has been reported that EZH2 depletion mediates histone H3K27 trimethylation and reduces the enrichment of DNMT1 on the miR-200b/a/429 promoter, thus abolishing transcriptional silencing of miRNAs and suppressing tumor growth in gastric cancer and glioblastoma [28].

The objective of this study was to investigate the regulatory mechanisms of miR-570-3p expression and its functional implications in PTC, focusing on the roles of DNA methylation and estrogen signaling. Specifically, the study aimed to assess how DNMT1 and DNMT3A, influenced by EZH2, affect the methylation and expression levels of miR-570-3p, and to determine the impact of estradiol (E2) on these processes and the subsequent effects on PTC tumor growth.

## Materials and methods

### Bioinformatics

The University of Alabama at Birmingham Cancer (UALCAN) data analysis Portal (https://ualcan.path.uab.edu/index.html) was used to analyze the expression of miR-570-3p in THCA patients. The Targetscan (https://www.targetscan.org/vert_80/), mirDIP (http://ophid.utoronto.ca/mirDIP/), and RNAInter (https://www.rna-society.org/rnainter3/) databases were used to analyze the interaction between hsa-miR-570-3p and DPP4. The methylation status of miR-570-3p was examined utilizing Methprimer database (http://www.urogene.org/methprimer/). The expression correlation between DNMT1, DNMT3A, DPP4, and EZH2 was predicted using the GEPIA database (http://gepia2.cancer-pku.cn/#index). The String (https://cn.string-db.org/) database was applied to analyze the interplay between EZH2, DNMT1, and DNMT3A.

### Patient tissue samples

Tumor tissues and adjacent normal tissues were collected from 30 PTC patients in The Tenth Affiliated Hospital of Southern Medical University (Dongguan People's Hospital) who underwent thyroidectomy without any radiotherapy or chemotherapy before the surgical operation. The tissues were identified by histopathological examinations and maintained at − 80 °C. Ethical approval was obtained from the Ethics Committee of The Tenth Affiliated Hospital of Southern Medical University (Dongguan People's Hospital), and all patients have signed informed consent. The patients were aged 27 ~ 82 years old, with 9 men and 21 women. There were 11 cases in the T1 stage, 10 in the T2 stage, 7 in the T3 stage, and 2 in the T4 stage. Regarding N classification, there were 13 N0 cases and 17 N1 cases.

### Cell culture and treatment

PTC cell lines (TPC-1 and GLAG-66) and normal cell lines (Nthy-ori3-1) were purchased from the Cell Bank of the Chinese Academy of Sciences. The cells were cultured in the RPMI-1640 medium (Gibco, NY) with 10% Fetal Bovine Serum (FBS, Thermo Fisher) and incubated in a humidified incubator at 37 °C and 5% CO_2_.

For Estradiol (E2) treatment assays, cells were incubated with 10 nM E2 [29], for 0.5, 2, and 6 h to evaluate its effect on various cellular processes. For the demethylation experiments, cells were cultured with 20 µM DNA methyl transferase inhibitor 5-Aza (Sigma-Adrich; USA) for 48 h. DMSO-treated cells were used as the control group.

### Cell transfection

The short hairpin RNAs (shRNAs) specifically targeting DNMT1, DNMT3A, DNMT3B, EZH2 and their corresponding negative control (NC) shRNAs, were constructed by GenePharma (Shanghai, China) and transfected into cells for silencing gene expression. For DPP4 overexpression, the pcDNA3.1-DPP4 and pcDNA3.1-NC vectors were designed by GenePharma. The miR-570-3p or NC mimics/inhibitors were synthesized by RiboBio (Shanghai, China). When the cell confluency reached 80%, the vectors and plasmids were transfected into TPC-1 and GLAG-66 cells using lipofectamine 3000 (Sigma-Aldrich), following the manufacturer's instructions. Cells were harvested 48 h after transfection for the following assays.

### RT-qPCR

Total RNA was extracted from PTC tissues and cells using the Trizol reagent (Invitrogen) according to the manufacturer’s protocol and then subjected to reverse transcription by a ReverTra Ace qPCR RT Kit (Toyobo, Japan). Next, the cDNA was used to detect the expression of target genes by qRT-PCR with FastStart Universal SYBR Green Master (Roche) on an ABI 7500 real-time PCR system (Applied Biosystems, USA). Relative expression of miR-570-3p, DPP4, Ezh2, DNMT1 and DNMT3A was analyzed using the 2^−ΔΔCt^ method. U6 or GAPDH served as the endogenous controls. The primer sequences used in this study are given in Table 1.

**Table 1:** Primer sequences used in this study

Primer name	Sequence (5’-3’)
miR-570-3p (forward)	AGTCCCTGCAGATATAGCTACAA
miR-570-3p (reverse)	AGACGTCAAGGCGGTAGGTAGTT
DPP4 (forward)	CTCCAGAAGACAACCTTGACCATTACAGAA
DPP4 (reverse)	TCATCATCATCTTGACAGTGCAGTTTTGAG
EZH2 (forward)	TTCCATGCAACACCCAACACAT
EZH2 (reverse)	TGGGCGTTTAGGTGGTGTCT
DNMT1 (forward)	GGGTCTCGTTCAGAGCTG
DNMT1 (reverse)	
DNMT3A (forward)	
DNMT3A (reverse)	GCAGGAATTCATGCAGTAAG
CCGCCTCTTCTTTGAGTTCTAC	
AGATGTCCCTCTTGTCACTAACG	
GAPDH (forward)	GGAAGCTTGTCATCAATGGAAATC
GAPDH (reverse)	TGATGACCCTTTTGGCTCCC
U6 (forward)	TGCTATCACTTCAGCAGCA
U6 (reverse)	GAGGTCATGCTAATCTTCTCTG

### Western blot

Total proteins were extracted from cells using the Radioimmunoprecipitation assay (RIPA) lysis buffer (Beyotime), and protein concentration was measured by an Enhanced bicinchoninic acid (BCA) Protein Assay Kit (Beyotime). Then protein samples were loaded onto 10% SurePAGE (GenScript) and electro-transferred onto PVDF membranes (Roche, Basel, Switzerland). The membranes were then blocked for 60 min at room temperature with 5% fat-free milk. Thereafter, membranes were incubated with the primary antibodies against DPP4, EZH2, DNMT1, and DNMT3A overnight at 4 °C. Next day, the membranes were washed three times with Tris-buffered saline Tween-20 before being treated with corresponding HRP-conjugated secondary antibodies at room temperature for 1 h. Finally, the ECL chemiluminescent detection reagent (Millipore, USA) was applied to visualize the proteins and analyzed using the ImageJ software.

### EdU assay

Cell proliferation was assessed using the EdU cell proliferation assay kit (Ribobio). Cells were seeded in 24-well culture plates and incubated with 100 μL of 50 μM EdU reagent (RiboBio, China) for 2 h. After fixing with 4% paraformaldehyde, cells were permeabilized with 0.1% Triton X-100 (Sigma-Aldrich). The nuclei of the cells were stained with DAPI (1 µg/mL) for 30 min. The images were observed under fluorescence microscopy (Olympus, Tokyo, Japan). We then determined the mean number of cells in three randomly chosen fields to evaluate cell proliferation.

### Colony formation assay

1 × 10^4^ cells/well were seeded in 6-well plates and grown for 2 weeks. Subsequently, the cell colonies were subjected to fixation using methanol (Sigma-Aldrich) for a duration of 30 min, after which they were stained with a 3% solution of crystal violet (Beyotime) for 20 min. Lastly, the colony in five randomly selected visual areas was calculated using a microscope (Olympus, Tokyo, Japan).

### Transwell assay

The 8-mm pore size Transwell chambers (Corning, Corning, NY) were applied to assess cell migration and cell invasion, without or with Matrigel, respectively. 1 × 10^4^ cells in serum-free medium were added to the upper chamber while the lower chamber was supplemented with complete medium. Following a 48-h incubation period, cells that moved or invaded the lower chamber membrane surface were fixed with 4% paraformaldehyde (Thermo Fisher) for 15 min and dyed with 0.1% crystal violet (Sigma-Aldrich). Subsequently, the quantification of migrating or invaded cells in the lower chambers was conducted under an inverted microscope (Olympus).

### Detection of cell apoptosis via flow cytometry assay

An Annexin V-FITC/Propidium Iodide (PI) Apoptosis Detection Kit (BD Biosciences, San Jose, CA) was used to measure cell apoptosis. In brief, the washed cells were resuspended in a 1X binding buffer. Next, 5 µl Annexin V-FITC and 5 µl PI were added to the cell suspension, and cells were kept at room temperature for 30 min in the dark. After washing, the cells were acquired using a BD FACS sorter (BD FACSVerse cytometer), and data was analyzed using FlowJo software.

### Fluorescent in situ hybridization (FISH) assay

FAM-labeled DPP4 or miR-570-3p probes were designed and synthesized by Foco (Guangzhou, China). In summary, cells were cultured on coverslips and fixated for 20 min with 4% paraformaldehyde. Following treatment with Pepsin (1% in 10 mmol/L HCl), the fixed cells were dehydrated continuously with ethanol. After dehydrating, the cells were combined with FISH probes in a hybridization buffer and incubated. Thereafter, the probe signals were determined using a Fluorescent In situ Hybridization Kit (Foco, Guangzhou, China) following the manufacturer’s protocol. DAPI was utilized to counterstain the nuclei of cells. Finally, a fluorescence microscope (Eclipse E6000; Nikon Corporation, Tokyo, Japan) was utilized to capture the images.

### Luciferase reporter assay

Wild type (WT) or mutated (MUT) DPP4 fragments complementary to miR-570-3p binding sites were subcloned into the pmirGLO luciferase vector (Promega, MA) for generating DPP4 WT/MUT. After co-transfection with luciferase vectors and indicated plasmids for 48 h, cells were analyzed by Luciferase Reporter System (Promega, Madison, WI) to measure the luciferase intensity.

### Methylation-specific PCR

Genomic DNA was extracted using a Genomic DNA Extraction Kit (Generay, China). Then, 1 µm of total genomic DNA was treated with sodium bisulfite in accordance with user guides of the EpiTect Fast DNA Bisulfite Kit (Qiagen, Germany). The methylation-specific PCR (MSP) of bisulfite-transformed DNA was performed using a nested, two-stage PCR method on an ABI 7500 real-time PCR system. 3% agarose gel electrophoresis was used to separate and amplify PCR products, which were further visualized with GelRed (Vazyme).

### RNA pull-down assay

This assay was performed with a Pierce Magnetic RNA–Protein Pull-Down Kit (Thermo Fisher Scientific) in accordance with user guides. Proteins from TCP-1 and GLAG-66 cells were mixed with the biotin-labeled RNA probe for miR-570-3p and then supplemented with streptavidin agarose beads (Life Technologies) at 4 °C for 1 h. The RNA–protein complexes were eluted using a biotin elution buffer and subsequently subjected to boiling in SDS for a duration of 10 min. Finally, coprecipitated RNAs were measured by RT-qPCR.

### Chromatin immunoprecipitation (ChIP) assay

ChIP assay was performed using the ChIP kit (Millipore, Billerica, MA) in accordance with user guidelines. TPC-1 and GLAG-66 cells were cross-linked with 1% formaldehyde at room temperature for 10 min, and the process was stopped by adding 125 mM of glycine. Subsequently, cells were subjected to the addition of lysis buffer, followed by the fragmentation of chromatin into DNA fragments ranging from 150 to 900 bp through sonication. Then, sonicated chromatins were immunoprecipitated with either a monoclonal anti-DNMT1, anti-DNMT3A (Abcam) or an isotype-matched control IgG Ab (Abcam). Following purification, qPCR amplification was performed on the precipitated complexes.

### Co-immunoprecipitation (Co-IP) assay

TPC-1 cells were lysed with RIPA lysis buffer, and then protein extracts were incubated with anti-EZH2, anti-DNMT3A, anti-DNMT1, or control anti-IgG at 4 °C overnight. Thereafter, the lysates were incubated for 6 h at 4 °C with Bioepitope R protein G/A agarose IP reagent beads (Bioworld Technology, Louis, CA) to precipitate protein antibody complexes. The complexes were then subjected to western blot assay.

### Animal study

Twenty-four female 6-week-old athymic BALB/C nude mice were purchased from The Tenth Affiliated Hospital of Southern Medical University (Dongguan People's Hospital). All animal-related experiments were approved by the Ethics Committee of The Tenth Affiliated Hospital of Southern Medical University (Dongguan People's Hospital). The transfected TPC-1 cells (1 × 10^6^) with miR-570-3p mimics/inhibitor or sh-NC were subcutaneously injected into mice to assess the effects of miR-570-3p on tumor growth. Each group contained 5 mice. After one week of cell injection, 3 mg/kg 5-Aza was injected intraperitoneally into mice [30]. Tumor volume (mm3) was calculated as follows every 7 days: tumor volume = length × width2 × 0.5. After 28 days of cell injection, mice were anesthetized with 3% isoflurane, euthanized, and tumors removed. Tumors were photographed and weighed and then stored at -80 °C for subsequent use.

### Hematoxylin and eosin (HE) staining

Tumor tissues were fixed with 4% paraformaldehyde, embedded in paraffin, and cut into 5-μm thick sections. Sections were stained with HE and then examined under an optical microscope (Olympus, Japan) to evaluate histopathological changes.

### Immunohistochemical (IHC) assay

The paraffin-embedded slices of mice tumor tissues were deparaffinized in xylene and rehydrated by fractional ethanol, followed by antigen retrieval, and blocking with goat serum. Subsequently, slices were incubated with the primary antibody against DPP4 or anti-Ki67 (Abcam) at 4 °C overnight. Next, slices were incubated with a secondary antibody (Abcam) for 2 h and stained with a DAB solution. Lastly, the images were photographed by a light microscope (Olympus, Japan).

### Statistical analysis

Data were analyzed with GraphPad Prism 6 software and are presented as the mean ± SD. The Student's t-test was employed to evaluate differences between the two groups, while one-way ANOVA with Tukey's post hoc test was used to compare multiple groups. The Spearman’s rank correlation coefficient was utilized for correlation analyses. All experiments were conducted at least three times. A p-value < 0.05 was considered statistically significant.

## Results

### MiR-570-3p expression is low and suppresses PTC cell malignant phenotypes

**Fig. 1:**
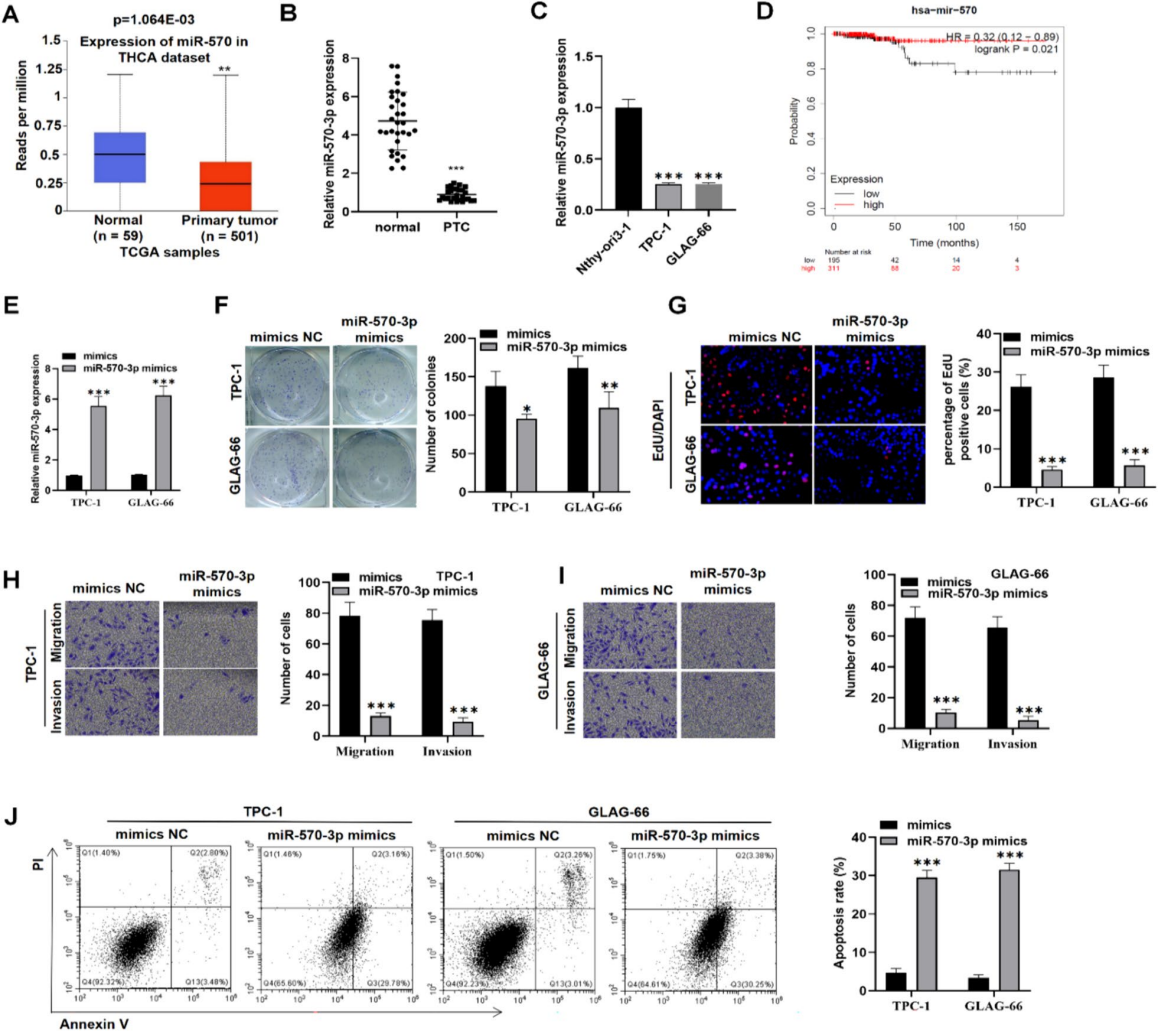
Expression and functional characteristics of miR-570-3p in PTC. **A** MiR-570-3p expression in THCA tissues (n = 501) and normal tissues (n = 59) was obtained using the UALCAN database. **B**, **C** RT-qPCR was performed to evaluate the gene expression level of miR-570-3p in 30 paired PTC tissues and adjacent non-tumor tissues (**B**) and in human PTC cell lines TPC-1, GLAG-66, and normal human thyroid cell line Nthy-ori 3–1 (**C**). **D** The Kaplan–Meier Plotter online tool was used to assess the association of miR-570-3p expression with the survival rate of PTC patients. **E** RT-qPCR was performed to measure the gene expression level of miR-570-3p in TPC-1 and GLAG-66 cells after transfection with NC mimics or miR-570-3p mimics for 48 h. **F**, **G** Colony formation and EdU experiments were conducted to measure cell proliferative capability in TPC-1 and GLAG-66 cells of NC mimics or miR-570-3p mimics groups. **H**, **I** Transwell assay was used to evaluate cell migratory and invasive capabilities in TPC-1 and GLAG-66 cells of NC mimics or miR-570-3p mimics groups. **J** Cell apoptotic ratio in NC mimics or miR-570-3p mimics-transfected TPC-1 and GLAG-66 cells was assessed using flow cytometry. *P < 0.05, **P < 0.01, ***P < 0.001

We first evaluated the expression profile of miR-570-3p in thyroid cancer (THCA) patients using the UALCAN (https://ualcan.path.uab.edu/index.html) database. We found that miR-570-3p expression was significantly lower in THCA patients (p = 1.064E-03, Fig. 1A). Subsequently, we assessed miR-570-3p expression in 30 tumor and adjacent normal tissues from PTC patients in our hospital (in-house). RT-qPCR results illustrated a decrease in miR-570-3p expression in PTC tissues compared to the adjacent normal tissues (Fig. 1B). We further confirmed this result in PTC cell lines (TPC-1 and GLAG-66) and a normal cell line (Nthy-ori3-1). As expected, miR-570-3p was downregulated in PTC cells compared to the control Nthy-ori3-1 cells (Fig. 1C). Kaplan–Meier analysis revealed that patients with low miR-570-3p expression had a lower survival rate, and vice versa (Fig. 1D). These results may indicate that miR-570-3p may play an important role in PTC pathogenesis.

Given the low expression of miR-570-3p in PTC, we speculated that miR-570-3p may function as a tumor suppressor gene in PTC. Thus, we conducted functional assays. Initially, miR-570-3p mimics were synthesized and transfected into TPC-1 and GLAG-66 cells, resulting in an upregulation of miR-570-3p levels post-transfection (Fig. 1E). Subsequently, colony formation and EdU assays were utilized to assess the proliferative capability of PTC cell. We found that miR-570-3p overexpression reduced colony formation capacity and decreased the percentage of EdU-positive cells, suggesting the suppression of PTC cell proliferation (Fig. 1F-G). Furthermore, transwell assays demonstrated that miR-570-3p overexpression decreased the number of migrated and invaded cells (Fig. 1H-I Additionally, flow cytometry analysis revealed a significant increase in the apoptotic rate in cells transfected with miR-570-3p mimics compared to the control NC mimics group (Fig. 1J). Collectively, these data confirm that miR-570-3p expression is low in PTC patients and that its upregulation inhibits malignant behaviors in PTC cells in vitro.

### MiR-570-3p targets DPP4 in PTC

**Fig. 2:**
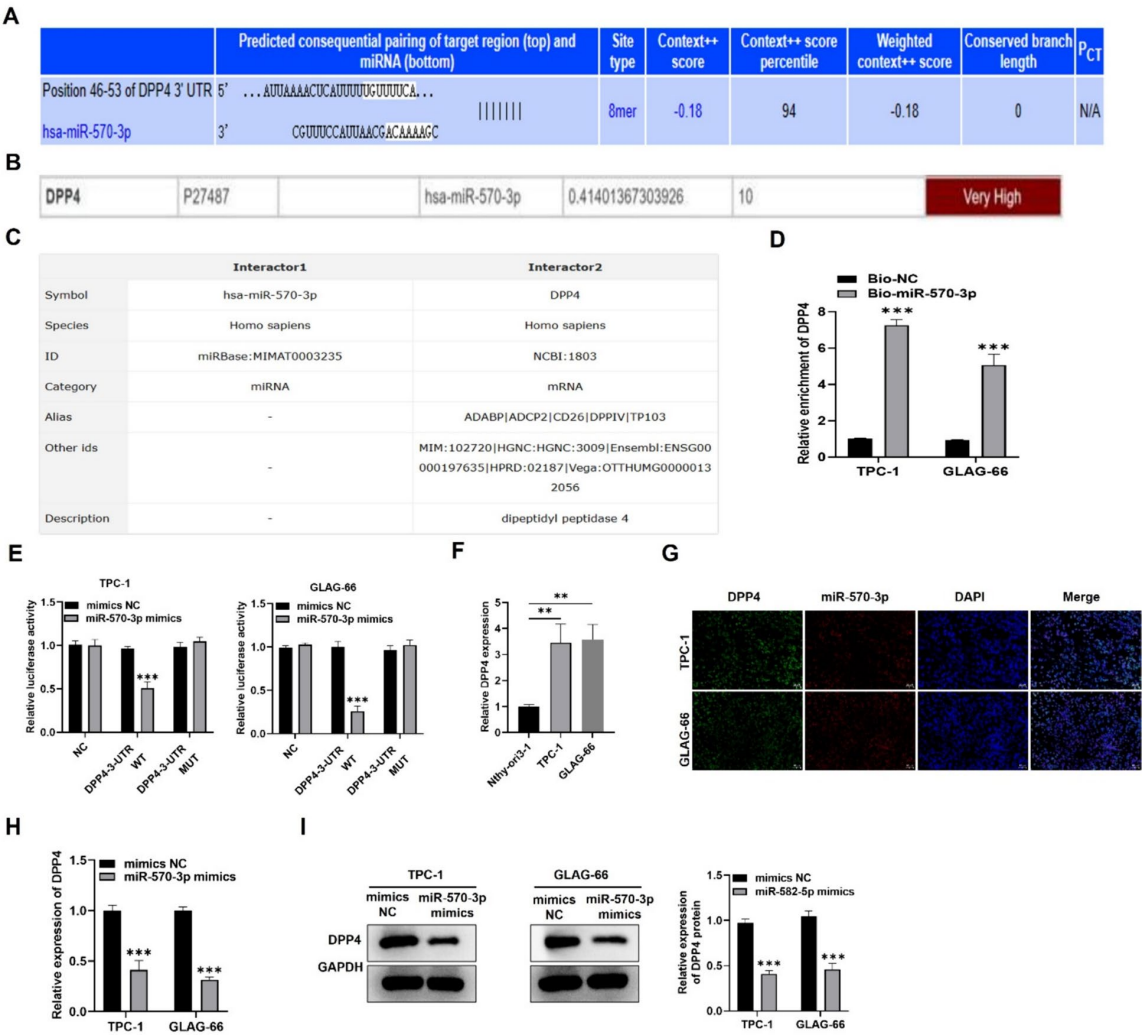
DPP4 is targeted by miR-570-3p in PTC. **A**–**C** The binding relationship of miR-570-3p and DPP4 was predicted through **a** Targetscan, **b** mirDIP, and **c** RNAInter databases. **D** RNA pulldown assay was conducted to assess the relative enrichment of DPP4 in the bio-miR-570-3p-formed complex in TPC-1 and GLAG-66 cells. The bio-NC group served as a negative control. **E** Luciferase reporter assay was performed to assess the luciferase activity of firefly plasmids containing wild type or mutated DPP4 3’UTR in TPC-1 and GLAG-66 cells after co-transfection of NC mimics or miR-570-3p mimics. (F) RT-qPCR was performed to measure DPP4 expression level in TPC-1 and GLAG-66 cells and control cell line Nthy-ori 3–1. **G** FISH assay was conducted to determine the co-location of miR-570-3p and DPP4 in TPC-1 and GLAG-66 cells. Scale bar = 100 μm. **H**, **I** RT-qPCR and western blot analysis results of DPP4 expression levels when miR-570-3p was overexpressed in TPC-1 and GLAG-66 cells. **P < 0.01, ***P < 0.001

In the next step, we aimed to identify the molecular target by which miR-570-3p mediates its function in PTC. Utilizing prediction tools from Targetscan (https://www.targetscan.org/vert_80/), mirDIP (http://ophid.utoronto.ca/mirDIP/), and RNAInter (https://www.rna-society.org/rnainter3/) databases, we found that hsa-miR-570-3p interacted with and targeted DPP4 (Fig. 2A-C). RNA pulldown assays showed that DPP4 was predominantly enriched in the pulldown products of biotinylated miR-570-3p in both TCP-1 and GLAG-66 cells (Fig. 2D). Subsequently, luciferase reporter assays were conducted to further confirm the interaction between miR-195-5p and DPP4 in PTC cells. Luciferase activity was notably reduced in DPP4-WT constructs when miR-570-3p was overexpressed in TPC-1 and GLAG-66 cells, while no significant changes were observed in the mutant constructs, indicating specific binding of miR-570-3p to DPP4 (Fig. 2E). We then evaluated the expression level of DPP4 in PTC and normal control cells. RT-qPCR demonstrated that DPP4 expression was higher in TCP-1 and GLAG-66 cells relative to the control (Fig. 2F). Next, we assessed the cellular localization of miR-570-3p and DPP4 in PTC cells. FISH assay demonstrated that miR-570-3p and DPP4 were predominately distributed in the cytoplasm of TCP-1 and GLAG-66 cells (Fig. 2G). Additionally, we assessed the mRNA and protein expression levels of DPP4 in TCP-1 and GLAG-66 cells transfected with miR-570-3p overexpression vectors. RT-qPCR and western blot assays demonstrated significant reductions in DPP4 mRNA and protein levels in cells transfected with miR-570-3p mimics, suggesting a negative regulatory effect of miR-570-3p on DPP4 expression (Fig. 2H-I). Taken together, these findings suggest that miR-570-3p may directly target DPP4 in PTC cells.

### DPP4 upregulation reverses the inhibitory functions of miR-570-3p in PTC

**Fig. 3:**
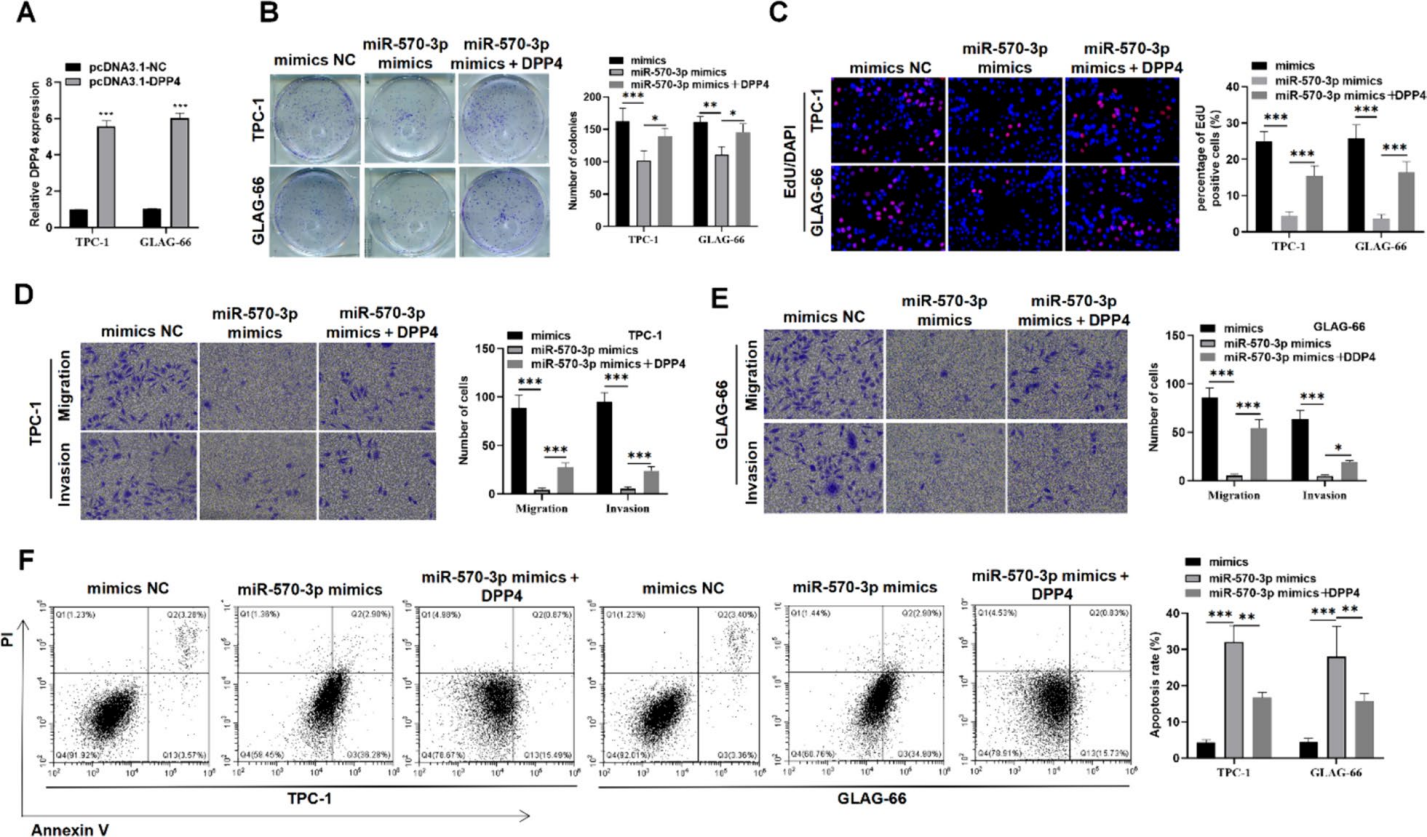
DPP4 upregulation abolishes the suppressive function of miR-570-3p in PTC. **A** RT-qPCR was conducted to validate the overexpression efficacy of pcDNA3.1-DPP4 in PTC cells. **B**, **C** Colony formation and EdU assays were utilized for assessing cell proliferative capability in the mimics NC group, miR-570-3p mimics group, and miR-570-3p mimics + DPP4 group in TPC-1 and GLAG-66 cells. **D**, **E** Transwell assay was used to measure TPC-1 and GLAG-66 cell migratory and invasive capabilities in the mimics NC group, miR-570-3p mimics group, and miR-570-3p mimics + DPP4 group. **F** The cell apoptotic ratio of TPC-1 and GLAG-66 cells in the indicated groups was assessed through flow cytometry. *P < 0.05, **P < 0.01, ***P < 0.001

To elucidate the regulatory mechanism of the miR-570-3p-DPP4 in PTC cellular processes, rescue experiments were conducted. DPP4 was overexpressed in TCP-1 and GLAG-66 cells through transfection with pcDNA3.1-DPP4. RT-qPCR analysis showed that DPP4 expression level was increased after transfection with pcDNA3.1-DPP4 (Fig. 3A). We observed that the reduced cell proliferative capacity, initially caused by upregulation of miR-570-3p, was restored upon DPP4 overexpression (Fig. 3B-C). Similarly, Similarly, transwell assays demonstrated that the upregulation of DPP4 significantly counteracted the suppressive effects of miR-570-3p mimics on the migratory and invasive capabilities of the cells (Fig. 3D-E). Additionally, the increased apoptosis rate observed in cells transfected with miR-570-3p mimics was mitigated by overexpressing DPP4 (Fig. 3F). These results demonstrate that upregulation of DPP4 can reverse the inhibitory effects of miR-570-3p in PTC.

### DNMTs bind to and methylate miR-570-3p promoter region

**Fig. 4:**
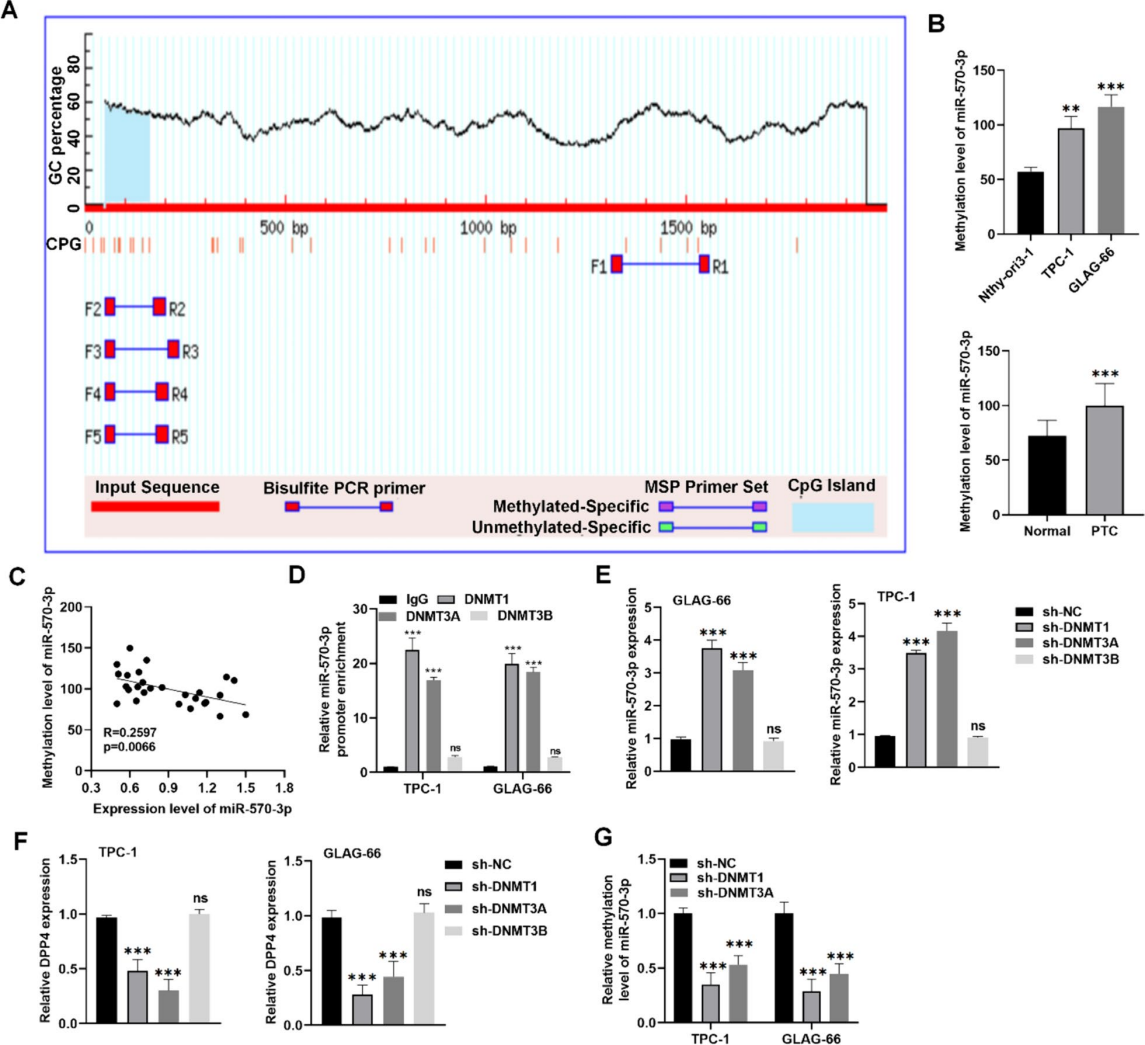
DNMTs regulate miR-570-3p promoter methylation. **A** Methprimer database was used to analyze the methylation site of the miR-570-3p promoter region. **B** MSP was used to detect the methylation level of miR-570-3p in PTC cells (upper panel) and in-house patient tissue samples (bottom panel). **C** Correlation analysis between the expression level and methylation level of miR-570-3p in PTC tissues. **D** ChIP assay was utilized to evaluate the interaction of the miR-570-3p promoter with DNMT1, DNMT3A, and DNMT3B in TPC-1 and GLAG-66 cells. **E**, **F** RT-qPCR analysis of miR-570-3p and DPP4 expression in TPC-1 and GLAG-66 cells transfected with sh-NC, sh-DNMT1, sh-DNMT3A, and sh-DNMT3B. **G** MSP was used to assess the miR-570-3p promoter methylation level in indicated groups. **P < 0.01, ***P < 0.001; ns means not significant

Epigenetic gene regulation is considered a key factor in the differential expression of miRNAs across various cancers. Therefore, we investigated the methylation status of the miR-570-3p promoter region. Using the Methprimer database, CpG islands were identified in the promoter region of miR-570-3p, which may indicate that the downregulation of miR-570-3p could be associated with promoter methylation (Fig. 4A). Subsequently, the experimental results further manifested the high methylation level of miR-570-3p promoter in PTC cells and tissues compared to the control cells and tissues (Fig. 4B). We also assessed the correlation between miR-570-3p methylation level and expression level using the TCGA database, which revealed a significant negative correlation in PTC tissues (Fig. 4C). DNA methyltransferases (DNMTs) play crucial functions in DNA methylation in the genome, thereby modulating target gene expression [31]. Therefore, we hypothesized that DNMTs might regulate the methylation of miR-570-3p. A ChIP assay confirmed that DNMT1 and DNMT3A, but not DNMT3B, bind to the miR-570-3p promoter (Fig. 4D). RT-qPCR analyses showed that knockdown of DNMT1 and DNMT3A significantly increased miR-570-3p expression and decreased DPP4 expression in TPC-1 and GLAG-66 cells compared to controls. However, silencing DNMT3B did not affect the expression of either miR-570-3p or DPP4 (Fig. 4E-F). Additionally, silencing DNMT1 and DNMT3A substantially reduced the methylation level of the miR-570-3p promoter in PTC cells (Fig. 4G). These results suggest that DNMT1 and DNMT3A suppress miR-570-3p expression by enhancing DNA methylation at its promoter region.

### EZH2 recruits DNMT1 and DNMT3A to inhibit miR-570-3p expression and upregulate DPP4

**Fig. 5:**
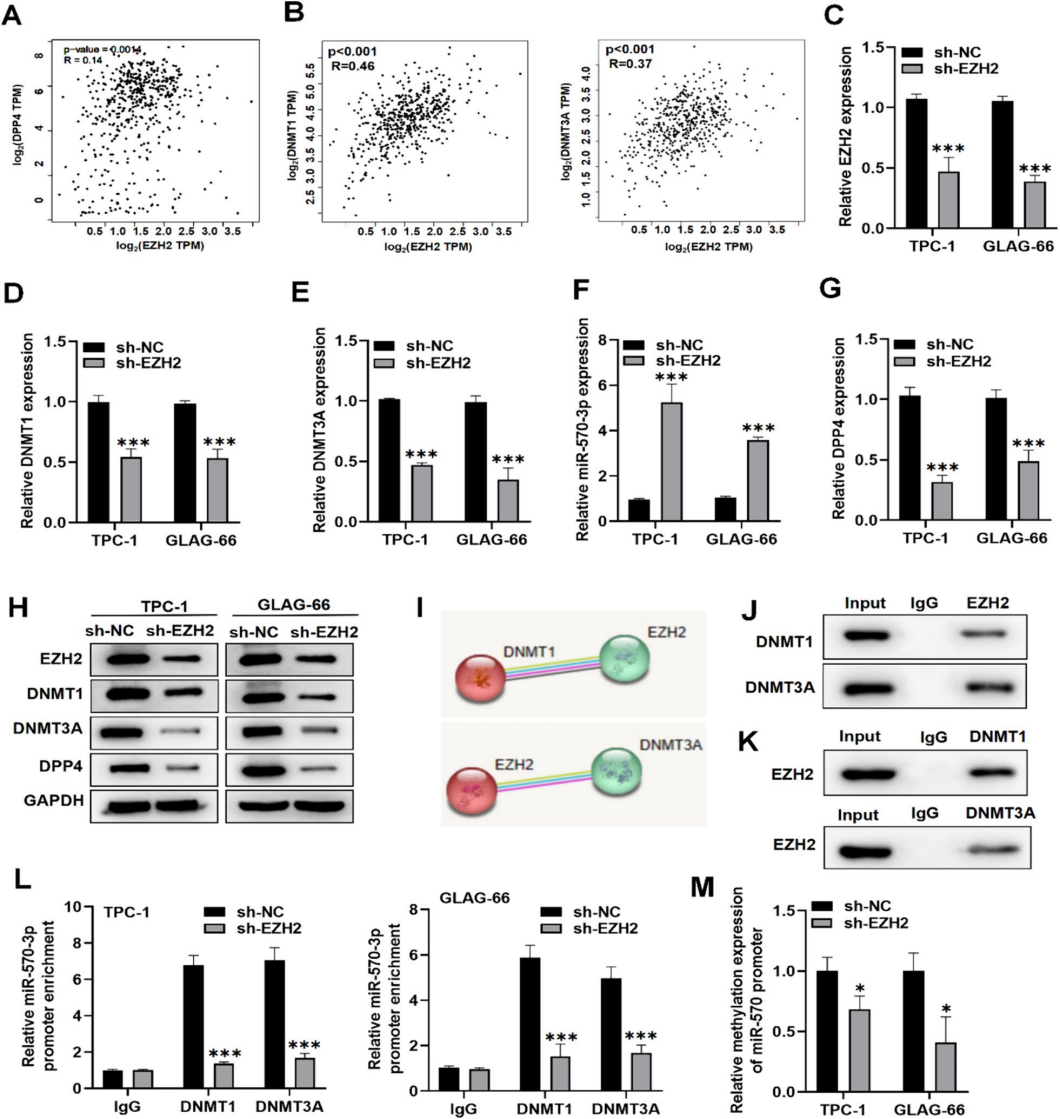
EZH2-recruited DNMT1 and DNMT3A inhibit miR-570-3p expression and upregulate DPP4. **A**, **B** The GEPIA database was used to explore the interaction of EZH2 with DPP4 (**A**), DNMT1 (**B**, left panel), and DNMT3A (**B**, right panel) in PTC. **C**–**G** RT-qPCR analysis of EZH2, DNMT1, DNMT3A, miR-570-3p, and DPP4 expression levels in TPC-1 and GLAG-66 cells transfected with sh-EZH2 or control sh-NC. **H** Western blot was performed to measure EZH2, DNMT1, DNMT3A, and DPP4 protein levels in TPC-1 and GLAG-66 cells transfected with sh-EZH2 or control sh-NC. **I** String database was used to evaluate the interaction relationship between EZH2 and DNMT1 or DNMT3A. **J**, **K** Co-IP assay was implemented to test the endogenous interplay of EZH2 and DNMT1 or DNMT3A. (L) ChIP assay was performed to determine the impact of EZH2 depletion on the binding ability of DNMT1 or DNMT3A with miR-570-3p promoter region. (M) MSP was used to determine the miR-570-3p promoter methylation level after silencing EZH2. *P < 0.05, ***P < 0.001

Studies have shown that EZH2 can recruit DNMTs to the promoter region, leading to the transcriptional inhibition of target genes [32, 33]. Since we found that DNMT1 and DNMT3A can regulate the expression level of both miR-570-3p and DPP4 in PTC by controlling methylation, we speculated that this regulation might be dependent on EZH2. To investigate this, we first examined the correlation between EZH2 and DPP4, DNMT1, and DNMT3A using the GEPIA database. We observed that EZH2 was positively correlated with DPP4, DNMT1, and DNMT3A in PTC (Fig. 5A-B). To further explore EZH2 role, we performed rescue experiments. RT-qPCR showed that EZH2 expression was downregulated in TCP-1 and GLAG-66 cells transfected with sh-EZH2 compared to the control sh-NC group (Fig. 5C). We discovered that EZH2 silencing notably reduced DNMT1, DNMT3A, and DPP4 expression levels, and promoted miR-570-3p expression level (Fig. 5D-G). Western blot analysis also showed that EZH2 inhibition decreased the protein levels of DNMT1, DNMT3A, and DPP4 (Fig. 5H). Data from the STRING database (https://cn.string-db.org/) further confirmed the interaction between EZH2 and DNMT1, as well as DNMT3A (Fig. 5I). Co-immunoprecipitation (Co-IP) results verified that EZH2 can endogenously bind to DNMT1 and DNMT3A (Fig. 5J-K). Additionally, the knockdown of EZH2 decreased the binding of DNMT1 and DNMT3A to the miR-570-3p promoter region (Fig. 5L) and reduced the methylation level of the miR-570-3p promoter (Fig. 5M). Collectively, these findings suggest that EZH2 recruits DNMT1 and DNMT3A to methylate the miR-570-3p promoter region, thereby inhibiting miR-570-3p expression.

### E2 regulates miR-570-3p promoter methylation via EZH2 recruited DNMT1 and DNMT3A and enhance DPP4 expression in PTC

**Fig. 6:**
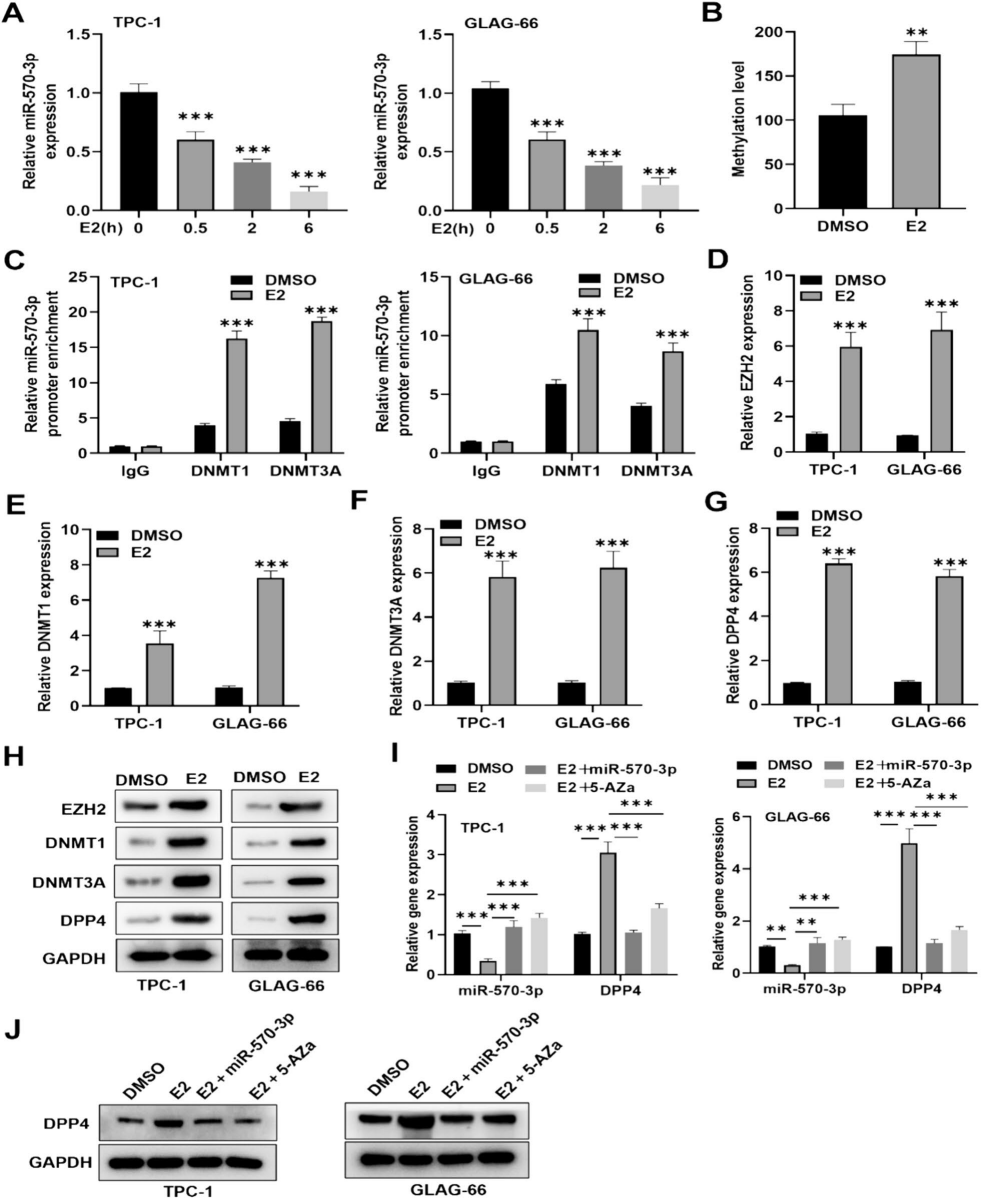
E2 regulates miR-570-3p promoter methylation. **A** RT-qPCR analysis of miR-570-3p expression under E2 or control DMSO treatment in TPC-1 and GLAG-66 cells. **B** MSP was used to detect the methylation level of the miR-570-3p promoter in DMSO or E2-treated TPC-1 and GLAG-66 cells. **C** ChIP assay was performed to determine the impact of E2 on the binding ability of miR-570-3p promoter region with DNMT1 or DNMT3A in TCP-1 and GLAG-66 cells. **D**–**G** RT-qPCR and **H** western blot analysis of EZH2, DNMT1, DNMT3A, and DPP4 expression levels in E2-treated or DMSO-treated TPC-1 and GLAG-66 cells. **I** RT-qPCR and **J** western blot analysis of DPP4 levels in the DMSO, E2, E2 + miR-570-3p mimic, and E2 + 5-AZa groups. **P < 0.01, ***P < 0.001

Next, we explored the effect of estradiol (E2) on the methylation of the miR-570-3p promoter. RT-qPCR results showed that E2 treatment decreased the expression levels of miR-570-3p in a time-dependent manner (Fig. 6A). We then evaluated the effect of E2 on miR-570-3p methylation level and found that E2 indeed promoted miR-570-3p methylation level in TCP-1 and GLAG-66 cells compared to the DMSO treated cells (Fig. 6B). Additionally, we confirmed the regulatory effects of E2 on DNA methyltransferases DNMT1 and DNMT3A. ChIP assay indicated that E2 treatment significantly enhanced the binding of DNMT1 and DNMT3A to the miR-570-3p promoter region in TCP-1 and GLAG-66 cells (Fig. 6C). Furthermore, we discovered that E2 treatment notably elevated the mRNA and protein levels of EZH2, DNMT1, DNMT3A, and DPP4 in PTC cells compared to the control treatment group (Fig. 6D-H). Subsequently, we examined the impacts of E2 on DPP4 level in TCP-1 and GLAG-66 cells either transfected with miR-570-3p mimic or treated with 5-AZa-DC (DNMT inhibitor). RT-qPCR analysis showed that miR-570-3p expression, markedly reduced by E2 treatment, was significantly restored by treatment with either miR-570-3p mimics or 5-AZa-DC in TCP-1 and GLAG-66 cells (Fig. 6I). Conversely, RT-qPCR and western blot analysis demonstrated that increases in DPP4 mRNA and protein levels induced by E2 treatment were reduced by overexpression of miR-570-3p or 5-AZa-DC treatment (Fig. 6I-J). Collectively, these findings uncover that E2 promotes the methylation of the miR-570-3p promoter through EZH2-mediated recruitment of DNMT1 and DNMT3A, which in turn facilitates DPP4 expression in PTC.

### MiR-570-3p methylation promotes PTC tumor growth in vivo

**Fig. 7:**
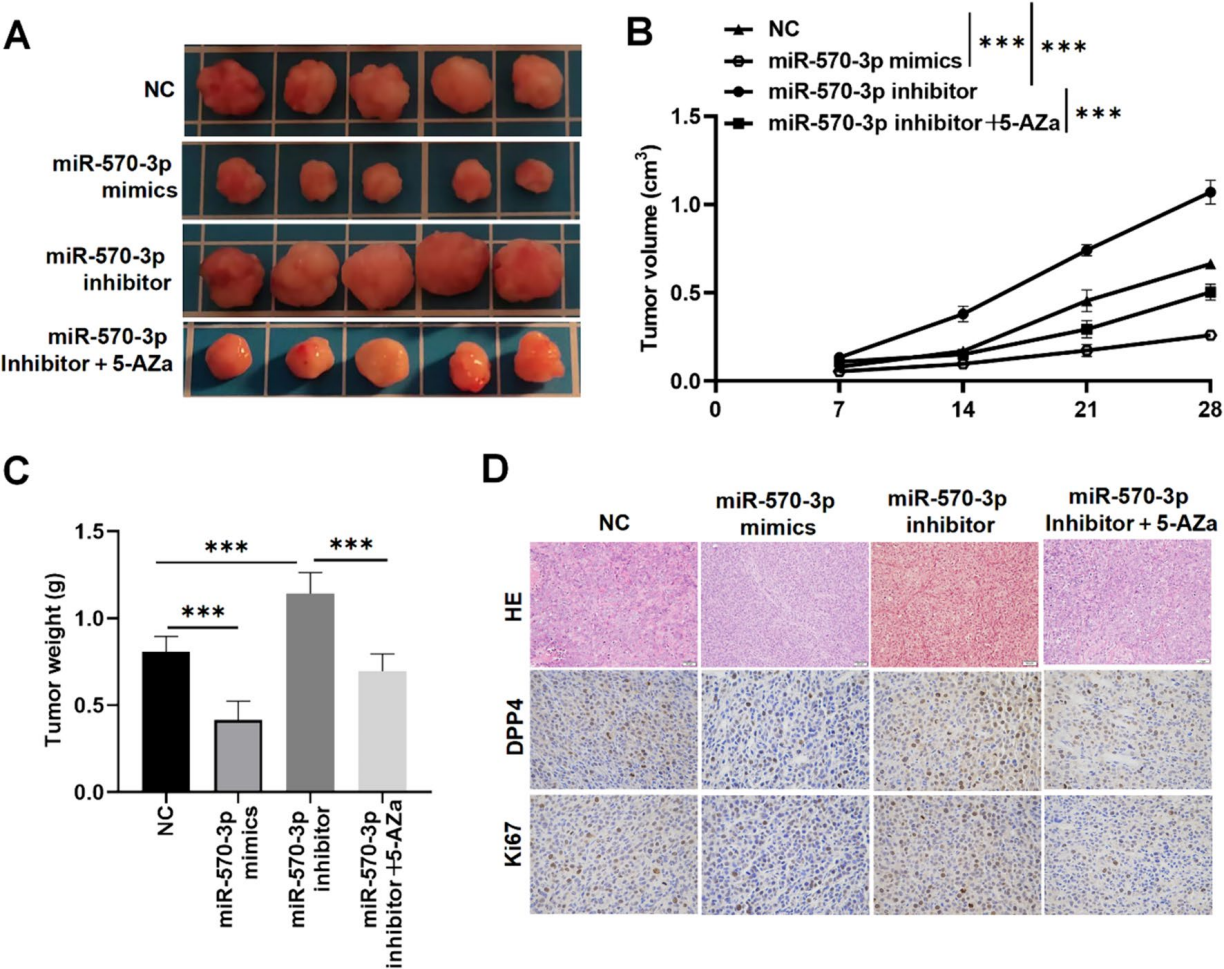
MiR-570-3p methylation contributes to PTC malignant progression in vivo. The transfected TPC-1 cells (1 × 10^6^) with miR-570-3p mimics/inhibitor or sh-NC were subcutaneously injected into mice for conducting tumor growth. **A** Representative tumor images were photographed from mice of four groups, including the NC group, the miR-570-3p mimics group, the miR-570-3p inhibitor group, and the miR-570-3p + 5-AZa inhibitor group. **B** Tumor volumes of mice in indicated groups were measured every 7 days. **C** The tumor weights of mice were measured on the 28th day after TPC-1 cells were injected into the mice in the indicated groups. **D** Representative images of HE staining and IHC staining of xenograft tumors of mice in four groups. Anti-DPP4 and anti-Ki67 were used for IHC. ***P < 0.001

Finally, we conducted xenograft tumor experiments to assess the function of miR-570-3p in PTC tumor progression in vivo. To this end, mice were xenotransplanted with TPC-1 cells (mimic NC group, miR-570-3p mimics group, miR-570-3p inhibitor group, and miR-570-3p inhibitor + 5-AZa group). We discovered that the tumor growth was slower in nude mice injected with TPC-1 cells transfected with miR-570-3p mimics compared to the control mimic NC group (Fig. 7A). Interestingly, inhibiting miR-570-3p significantly promoted tumor growth. Of note, when these mice were injected with 5-Aza, a DNMT inhibitor, it prominently lessened the tumor growth compared to the miR-570-3p inhibitor group (Fig. 7A). Similarly, the tumor volume and weight in the miR-570-3p mimics group were significantly decreased, whereas inhibition of miR-570-3p resulted in a significant increase in both tumor volume and weight (Fig. 7B-C). As expected, when the mice in the miR-570-3p inhibitor group were injected with 5-Aza, tumor volume, and weight were suppressed (Fig. 7B-C). Subsequently, we evaluated DPP4 and Ki67 expression levels in these mice. Results demonstrated that DPP4 and Ki67 expression in tumor tissues was suppressed by miR-570-3p overexpression and elevated by miR-570-3p knockdown (Fig. 7D). Treatment with 5-AZa reversed the effects of miR-570-3p knockdown on tissue structure and on DPP4 and Ki67 expression levels (Fig. 7D). Taken together, these findings imply that miR-570-3p methylation accelerates PTC development in vivo.

## Discussion

Tumor metastasis is the main risk factor for poor prognosis in PTC [34]. Several studies have shown that miRNAs are closely related to oncogenesis and can serve as diagnostic and prognostic biomarkers [35]. At present, a large number of miRNAs are dysregulated in PTC, playing key regulatory roles; examples include miR-4443 [36], miR-205-5p [37], and miR-451a [38]. MiR-570-3p is involved in various biological processes and has been proven to play a regulatory function in malignant tumors [18, 21, 39]. In agreement with previous reports, we confirmed that miR-570-3p is downregulated in PTC tissues and cells, and its elevated expression is associated with a better survival rate. Upregulation of miR-570-3p suppressed proliferative, migratory, and invasive capabilities of PTC cells and expedited apoptosis. Additionally, miR-570-3p overexpression alleviated PTC tumor growth in mice, whereas miR-570-3p inhibition had the opposite effect. These results confirm the tumor suppressor role of miR-570-3p in PTC.

DPP4 belongs to the prolyl oligopeptidase serine protease family [40]. MiRNAs are known to primarily suppress gene expressions by binding to the 3’UTR of target transcripts [41]. Through bioinformatics analysis, we identified DPP4 as a potential target gene of miR-570-3p and hypothesized that miR-570-3p may suppress DPP4 expression to restrain the progression of PTC. Studies have shown that DPP4 is upregulated in various tumors and contributes to cancer development [42]. For example, DPP4 upregulation can facilitate distant metastasis of esophageal adenocarcinoma and colorectal cancer [43, 44]. DPP4 is shown to promote tumor metastasis in lung adenocarcinoma [45]. Importantly, evidence has manifested that DPP4 overexpression in PTC is closely associated with tumor invasion and may serve as a potential prognostic marker for PTC [46]. Herein, we demonstrated that miR-570-3p targets DPP4 in PTC cells and negatively regulates DPP4 expression. Moreover, DPP4 overexpression reversed the suppressive functions of miR-570-3p on PTC progression. Therefore, we confirm that miR-570-3p exerts a tumor-suppressive effect in PTC by inhibiting DPP4.

Several factors may contribute to a reduction in miRNA production in human cancers, such as alterations in miRNA processing and epigenetic dysregulation [47]. Anomalous epigenetic modulation can lead to changes in gene expression and the malignant transformation of cells [48]. DNA methylation, which involves the addition of a methyl group at the 5-carbon position of the cytosine ring within CpG dinucleotides in a promoter, is a prevalent epigenetic mechanism critical for tumor growth and metastasis [49]. A report suggested that DNA methylation alterations are associated with prognosis in well-differentiated thyroid lesions [50]. Several studies have proven that miRNAs can be epigenetically silenced in tumors via promoter methylation [51, 52]. Methylation-mediated miR-125a-5p silencing expedites breast cancer development via activating autophagy [53]. Methylation-mediated miR-204 silencing facilitates lymph metastasis in PTC [54]. It has been reported that DNMT1-mediated epigenetic silencing of miR-200b/a/429 facilitates the development of gastric cancer and glioblastoma [28]. Wu et al. have reported that inhibition of miR-199a-3p by promoter methylation contributes to PTC aggressiveness by regulating DNMT3A [55]. In our study, we identified CpG islands in the miR-570-3p promoter and confirmed high levels of miR-570-3p methylation in PTC. A previous study revealed that demethylation-activated miR-570-3p suppresses LCMR1 and ATG12, contributing to the anti-metastatic effects of metformin in human osteosarcoma [19]. Thus, we studied the interaction between miR-570-3p and DNMTs in PTC. DNMT1 and DNMT3A were found to negatively regulate miR-570-3p expression and positively influence DPP4 expression. They can bind to the miR-570-3p promoter, and their deficiency reduced the methylation levels of miR-570-3p. Thus, we demonstrate that DNMTs suppress miR-570-3p through promoter methylation, thereby enhancing its expression in PTC.

Studies have indicated that EZH2 is upregulated in PTC, and its expression positively correlates with tumor staging [56]. Herein, we found a notable positive association between EZH2 and DNMT1, DNMT3A, and DPP4 in PTC cells. EZH2 can interact endogenously with DNMT1 and DNMT3A, and knocking down EZH2 weakens the affinity between miR-570-3p and DNMTs. Previous reports have shown that DNMT1, together with EZH2, contributes to the transcriptional repression of the miR-200 family members, and EZH2 depletion reduces DNMT1 presence on the miR-200b/a/429 promoter in cancer cells [28]. Furthermore, miR-142-3p has been shown to be epigenetically inhibited by EZH2-recruited DNMT1, suppressing the metastasis of nasopharyngeal carcinoma [57]. Moreover, it has been confirmed that SChLAP1 facilitates prostate cancer progression by interacting with EZH2 to mediate DNA methylation of various miRNAs on chromosome 5 with a DNMT3A-feedback loop [58]. Therefore, we believe that EZH2 and DNMTs may collectively exert a transcriptional inhibition function on miR-570-3p in PTC.

Studies have confirmed that estrogen is a potent developmental factor for PTC. Upregulation of ER-β facilitates cancer stem-like properties in PTC [59]. E2-induced cell proliferation of PTC is mediated via ER-α and ER-β [60]. Furthermore, it has been reported that estrogen reduces FAM111B expression by DNMT3B methylation and facilitates tumor growth in PTC [61]. MiR-148a regulates the expression of the estrogen receptor through DNMT1-mediated DNA methylation in breast cancer cells [62]. Additionally, it has been reported that EZH2 is required for estrogen-induced cell proliferation in ER-positive breast cancer, and E2 can upregulate EZH2 levels via ERα signaling [63]. In our study, we found that E2 treatment suppressed miR-570-3p expression in PTC cells, increased its methylation level, and elevated EZH2, DNMT1, DNMT3A, and DPP4 expression levels. Furthermore, miR-570-3p overexpression or treatment with 5-Aza counteracted the promoting effects of E2 on DPP4 expression. Treatment with 5-Aza also abolished the promoting effect of miR-570-3p deficiency on tumor growth. Therefore, we conclude that E2 suppresses miR-570-3p expression in PTC by regulating methylation.

Taken together, this study confirms that estrogen regulates the EZH2/DNMTs/miR-570-3p/DPP4 signaling pathway to promote PTC progression. These findings may provide novel therapeutic targets for PTC.

## Data Availability

The datasets generated during and/or analyzed during the current study are available from the corresponding author upon reasonable request.
